# Differences in Finger Dexterity in Patients With Mild and Moderate Alzheimer's Disease—A Study of Cognitive Function by Disease Severity

**DOI:** 10.1002/brb3.70403

**Published:** 2025-03-09

**Authors:** Shota Suzumura, Aiko Osawa, Junpei Sugioka, Masaki Kamiya, Yuko Sano, Akihiko Kandori, Tomohiko Mizuguchi, Yoshiharu Uchida, Hitoshi Kagaya, Izumi Kondo

**Affiliations:** ^1^ Faculty of Rehabilitation, School of Health Sciences Fujita Health University Toyoake Japan; ^2^ Department of Rehabilitation Medicine National Center for Geriatrics and Gerontology Obu Japan; ^3^ Research & Development Group, Healthcare Innovation Center Hitachi, Ltd. Kokubunji Japan; ^4^ Research & Development Group, Center for Exploratory Research Hitachi, Ltd Kokubunji Japan; ^5^ New Business Producing Division, Business Development Dept. Maxell, Ltd. Yokohama Japan

## Abstract

**Aim:**

This study aimed to estimate the relationship between finger function and cognitive function in patients with Alzheimer's disease (AD).

**Methods:**

Patients diagnosed with AD at the Outpatient Center for Comprehensive Care and Research on Memory Disorder of the National Center for Geriatrics and Gerontology underwent a 15‐s bimanual alternating tapping task to measure finger movements. After finger movement measurements, we classified the severity of AD into mild and moderate and compared the finger movements. The Mann–Whitney *U* test and effect size were used to compare parameter values between the two groups (mild and moderate AD), and the calculated *p* values were corrected using the Bonferroni method. The Spearman rank correlation coefficient was calculated to determine the association between finger parameters and cognitive function (Mini‐Mental Examination [MMSE]).

**Results:**

Data from 163 patients with AD were analyzed. When comparing finger parameters between the mild AD (64 individuals) and moderate AD (99 individuals) groups, the moderate AD group demonstrated fewer taps (*p* = 0.005; *r* = 0.22) and a longer interval between taps with the thumb and index finger (*p* = 0.007; *r* = 0.21) than the mild AD group. The correlation between the MMSE score and finger function was weakly positive for the number of taps and weakly negative for the average of tapping interval.

**Conclusions:**

These parameters reflect the decline in finger function associated with the advanced stages of dementia and may help assess the severity of AD. Additionally, these findings may have clinical utility in assessing the severity of AD, potentially enhancing diagnostic accuracy for differentiating stages of AD.

## Graphical abstract

This study examines the relationship between finger dexterity and cognitive function in patients with Alzheimer's disease (AD). The results indicate that patients with moderate AD demonstrate significantly fewer taps and longer tapping intervals compared to those with mild AD, suggesting a progressive decline in motor function. These findings highlight the potential of finger‐tapping assessments as a complementary tool for evaluating AD severity.

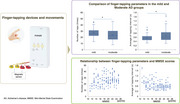

## 1 Introduction

Currently, over 55 million people worldwide have dementia, with nearly 10 million new cases annually (World Health Organization). In Japan, the number of patients with dementia is expected to exceed 7.4 million by 2030, mainly because of the progression from mild cognitive impairment (MCI) to dementia (Ministry of Health, Labour and Welfare, Health and Welfare Bureau for the Elderly). According to the Comprehensive Survey of Living Conditions, dementia is the primary reason for the need for care (Welfare, MoH 2019). The increase in dementia incidence has become a major social problem, with expectations for further increases in the future. Consequently, the social demand for early diagnosis of dementia is increasing.

Dementia occurs when brain function gradually declines and cognitive functions, such as thinking, judgment, memory, and language ability, are impaired. Neuropathological changes cause disease progression over several decades before the onset of dementia symptoms (Kivimäki and Singh‐Manoux 2018). Alzheimer's disease (AD), one of the most common causes of dementia, constitutes 80% of all cases and predominantly affects older adults (Crous‐Bou et al. 2017). The progression of AD varies substantially from person to person (Suh et al. 2004); however, AD symptoms generally begin with mild memory impairment, followed by a gradual decline in other cognitive functions. In addition, patients with AD show impairments in instrumental activities of daily living (IADL), such as meal preparation and shopping, and in later stages of the disease, in basic activities of daily living, such as eating and bathing (Suh et al. 2004; Slachevsky et al. 2019). The progression and severity of dementia are often measured using the Mini‐Mental Examination (MMSE) (Folstein et al. 1975), which evaluates overall cognitive function; Clinical Dementia Rating (CDR) (Hughes et al. 1982), which assesses the degree of cognitive function based on the activities of daily living (ADL) situation; and Functional Assessment Staging (FAST) (Reisberg 1988). Although the outcomes of these measures and clinical progression are not necessarily linear, patients with dementia have been reported to decline by an average of 3–3.5 points on the MMSE each year (Nourhashémi et al. 2008; Han et al. 2000; Adak et al. 2004; Clark et al. 1999).

Recent studies have shown that motor dysfunction occurs before cognitive problems appear, which could be a clinical indicator for predicting the signs of dementia (Buracchio et al. 2010). Several aspects of motor function are closely related to cognitive function, with many studies focusing specifically on lower‐extremity motor functions. For example, the MCI group showed a decreased dual‐task gait performance compared with the group with normal cognitive function (Wang et al. 2023). The Canadian Consensus Conference on Diagnosis and Treatment of Dementia also recommends the inclusion of motor function assessment in dementia testing, given strong evidence supporting its efficacy in detecting cognitive impairment and dementia risk in older adults (Montero‐Odasso et al. 2020). Furthermore, studies have demonstrated that patients with AD exhibit decreased finger dexterity and lack of accuracy compared with controls regarding finger function (Fritz et al. 2016; Yu and Chang 2016). We also detected a decrease in the number of taps and coordinated movements of both fingers in patients with cognitive decline (Suzumura et al. 2022). While the relationship between motor and cognitive functions in patients with dementia is gradually becoming clearer, differences in finger function based on dementia severity have not been reported. It is crucial to investigate finger function differences between mild and moderate AD to understand the extent to which motor dysfunction is related to the severity of cognitive decline. By analyzing finger function within groups categorized by cognitive performance, we may identify specific motor impairments associated with different stages of AD. Thus, monitoring the progression and severity of motor impairments in dementia could enable tracking of the disease progression and facilitate early detection.

This study aimed to estimate the relationship between finger function and cognitive function in patients with AD. Considering recent findings on motor dysfunction in cognitive disorders, we hypothesized that motor skills, specifically finger dexterity, would be poorer in moderate AD than in mild AD. This hypothesis is anticipated to correlate with the severity of cognitive impairment determined through standardized assessments such as the MMSE.

## 2 Methods

### 2.1 Study Design and Population

This cross‐sectional study was conducted at the National Center for Geriatrics and Gerontology in Obu, Japan. The participants were patients diagnosed with AD at our outpatient Center for Comprehensive Care and Research on Memory Disorders. The diagnostic criteria for AD were based on those specified by the National Institute on Aging—Alzheimer's Association (Mckhann et al. 2011). The diagnostic evaluation included (1) a medical examination by a dementia specialist, (2) an informant interview, (3) physical and cognitive evaluation, (4) head magnetic resonance imaging and single‐photon emission computed tomography by a clinical laboratory technician, and (5) electrocardiography and blood tests. Finally, the dementia specialist made a comprehensive judgment based on the aforementioned results and diagnosed the patient with AD. The selection criteria for the participants were as follows: (1) older than 65 years of age, (2) right‐handedness, and (3) ability to understand the measurement method. Patients with a history of stroke, Parkinson's disease, other neurodegenerative diseases, tremors, depression, or finger dexterity disorders that interfered with finger movement measurements were excluded.

Cognitive function was assessed using the MMSE (Folstein et al. 1975), with patients classified according to the MMSE score ranges defined in Perneczky et al.’s study: (1) 21–25 points indicate mild AD, (2) 11–20 points indicate moderate AD, and (3) 0–10 points indicate severe AD (Perneczky et al. 2006). Individuals with MMSE scores of ≥26 points who were not included in the previous study by Perneczky et al. were excluded (Perneczky et al. 2006) (details of the MMSE are described in Section 2.3 “Evaluation tool”). Before the finger‐tapping movement measurement, patients with AD were asked to complete a medical questionnaire to obtain background information related to dementia, including date of birth, dominant hand, medical history, and ADL situation. In instances where patients found it challenging to complete the medical questionnaire, an accompanying family member would do so.

**FIGURE 1:**
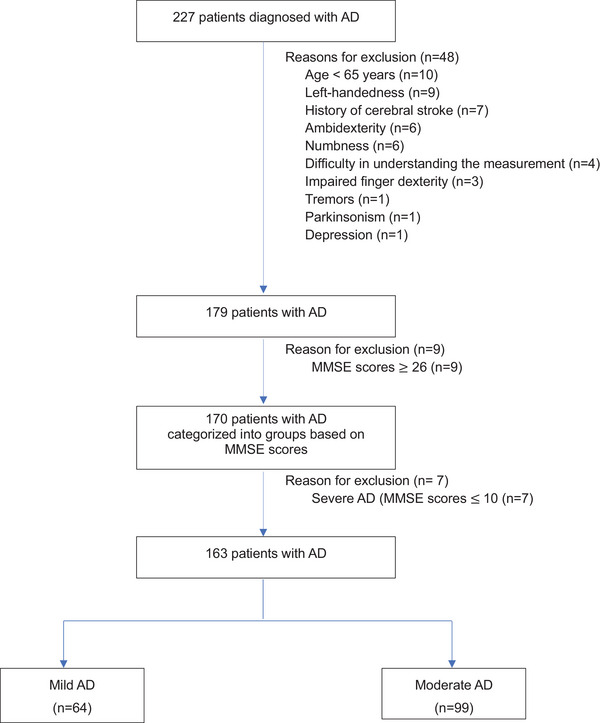
Study flowchart. AD, Alzheimer's disease; MMSE, Mini‐Mental Examination.

Figure 1 shows the study flowchart. A total of 227 patients were diagnosed with AD. On the basis of the selection and exclusion criteria, we included 163 participants in this study. According to their MMSE scores, the participants were classified as having mild (*n* = 64) or moderate (*n* = 99) AD. Table 1 shows the participants’ characteristics.

**TABLE 1:** Participant characteristics.

Characteristic	Mild AD (*n* = 64)	Moderate AD (*n* = 99)	*p* value
Age (years)	79.1 (5.4)	79.9 (6.4)	0.687
Male/Female	24/40	25/74	0.197
Education (years)	10.1 (2.0)	10.6 (2.4)	0.101
Grip Right (kg) Left (kg)	19.9 18.8	18.8 17.7	0.503 0.492
Timed Up & Go test(s)	11.6 (3.1)	13.5 (4.2)	0.002*
MMSE (points)	23 (22–24)	16 (14–19)	≤0.001*
DBD (points)	11 (6–16)	15 (9–21)	0.058
GDS (points)	2 (1–4)	2 (1–5)	0.561
VI (points)	9 (9–10)	9 (8–10)	0.191
J‐ZBI (points)	16 (7–25)	17 (11–27)	0.426
BI (points)	100 (95–100)	100 (90–100)	0.009*

*Note*: Data are presented as mean (SD) or median (quartiles). Statistically significant values (*p* < 0.05) are indicated with an asterisk (*).

Abbreviations: AD, Alzheimer's disease; BI, Barthel Index; DBD, Dementia Behavior Disturbance Scale; GDS, Geriatric Depression Scale; J‐ZBI, Japanese version of the Zarit caregiver burden; MMSE, Mini‐Mental State Examination; s, seconds; SD, standard deviation; VI, Vitality Index.

A power analysis was conducted using the G*Power software to determine the required sample size. Based on an anticipated effect size of 0.5, an alpha level of 0.05, and a power of 0.80, the analysis indicated that 134 participants would be required to detect significant differences in finger function between patients with mild and moderate AD. Our study included 163 participants, exceeding the minimum requirement for adequate statistical power.

### 2.2 Procedures

**FIGURE 2:**
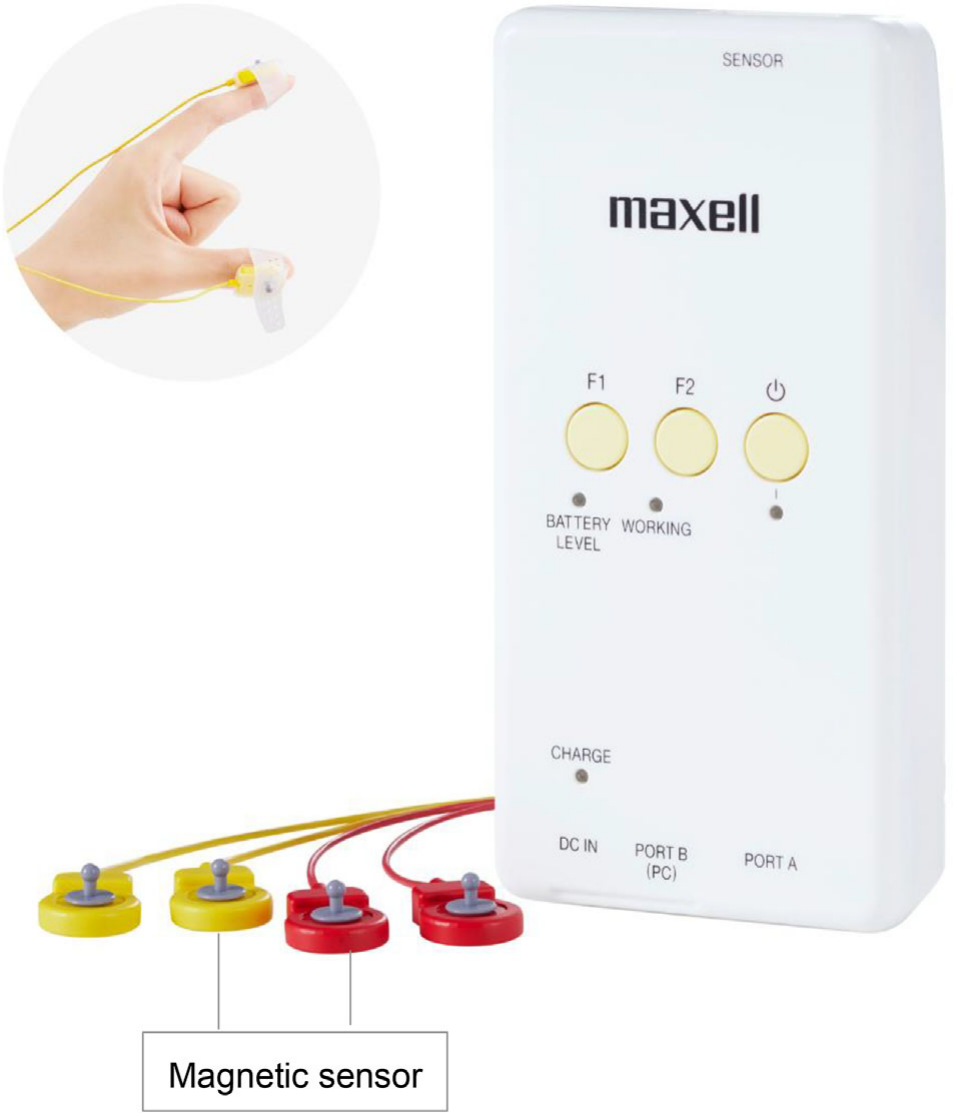
UB‐2 measuring devices and finger‐tapping movement. UB‐2 is a nonmedical device that uses magnetic sensors for safe and accurate measurements. During finger tapping, magnetic sensors were attached to patients’ left and right thumbs and index finger. Alternate tapping with both hands: Perform finger tapping alternately with the left and right hands as fast as possible for 15 s, keeping the distance between the thumb and index finger at 3–4 cm.

A UB‐2 magnetic sensing finger‐tapping device (Maxell, Tokyo, Japan) was used as the measuring device (Figure 2). The reliability of finger‐tapping devices has been evaluated for the following three types of reproducibility: when measurements were taken at different times, with different devices, and during inter‐rater testing. The reproducibility has been reported to be high (Sano et al. 2011). Magnetic sensing uses a transmission coil on the index finger and flows a sinusoidal current, generating a magnetic field. Based on the principle of electromagnetic induction, a reception coil on the thumb detects a magnetic field. According to the detected electric voltage, the distance between the coils was converted to finger intervals (Kandori et al. 2004). In our previous study, compared with the control group, cognitively impaired individuals had significantly poorer finger function in the both‐handed alternating tapping task than in the one‐handed tapping task (Suzumura et al. 2022). Furthermore, a significant difference was found in the left hand in the two‐handed alternating tapping task. Therefore, on the basis of previous studies, we analyzed the left hand in the both‐handed alternating tapping task. In this study, we analyzed four parameters based on our previous study (Suzumura et al. 2022) and the previous study by Koppelmans et al. (Koppelmans et al. 2023; Koppelmans et al. 2024). These parameters included the number of taps (indicating the number of times tapping was performed during the measurement period), average of tapping interval (indicating the average time from one tap to the next and the tapping speed), the standard deviation of the intertapping interval (indicating how much the tapping interval varied), and number of freezing calculated from acceleration (indicating the number of small freezing movements other than tapping). The magnetic sensors were placed on the participants’ thumbs and index fingers of both hands using an elastic band. The finger‐tapping task consisted of alternating finger tapping with both hands (left‐ and right‐hand tapping), performed as fast as possible for 15 s while maintaining the amplitude at 3–4 cm. The details of the measurement environment are shown in Figure S1 (Suzumura et al. 2022).

### 2.3 Evaluation Tool

Cognitive function was assessed using the MMSE (Folstein et al. 1975), as previous studies have demonstrated the reliability and validity of MMSE (Tombaugh and McIntyre 1992). The MMSE is comprised of 11 short‐form cognitive probes and is used to assess the progression of dementia in many clinical situations. The duration was approximately 10–15 min. We also used the Japanese Version of the Dementia Behavior Disturbance (DBD) scale to assess behavioral abnormalities associated with dementia (Mizoguchi et al. 1993), Japanese Version of the Zarit Caregiver Burden Interview (J‐ZBI) to assess care burden (Arai et al. 1997), Geriatric Depression Scale (GDS) to assess depression (Yesavage and Sheikh 1986), Vitality Index (VI) to assess motivation (Toba et al. 2002), and Barthel Index (BI) to assess the ability to perform ADL (Mahoney and Barthel 1965). In addition, a physical assessment was conducted using the Grip and Timed Up & Go (TUG) test.

### 2.4 Statistical Analysis

We used the Mann–Whitney *U* test and effect size to compare the differences in finger parameter values between the two groups (mild and moderate AD). An alpha threshold of 0.0125 was applied to account for multiple comparisons using a Bonferroni correction for the four outcome measures (0.05/4). This adjustment minimized the risk of Type I errors. For items that showed further significance, Spearman rank correlation coefficients were calculated to determine the relationship between finger parameters and cognitive function (MMSE score). Statistical analyses were performed using SPSS (version 26.0; IBM Japan, Tokyo, Japan).

## 3 Results

### 3.1 Comparison of Finger‐Tapping Parameters in the Mild and Moderate AD Groups

**FIGURE 3:**
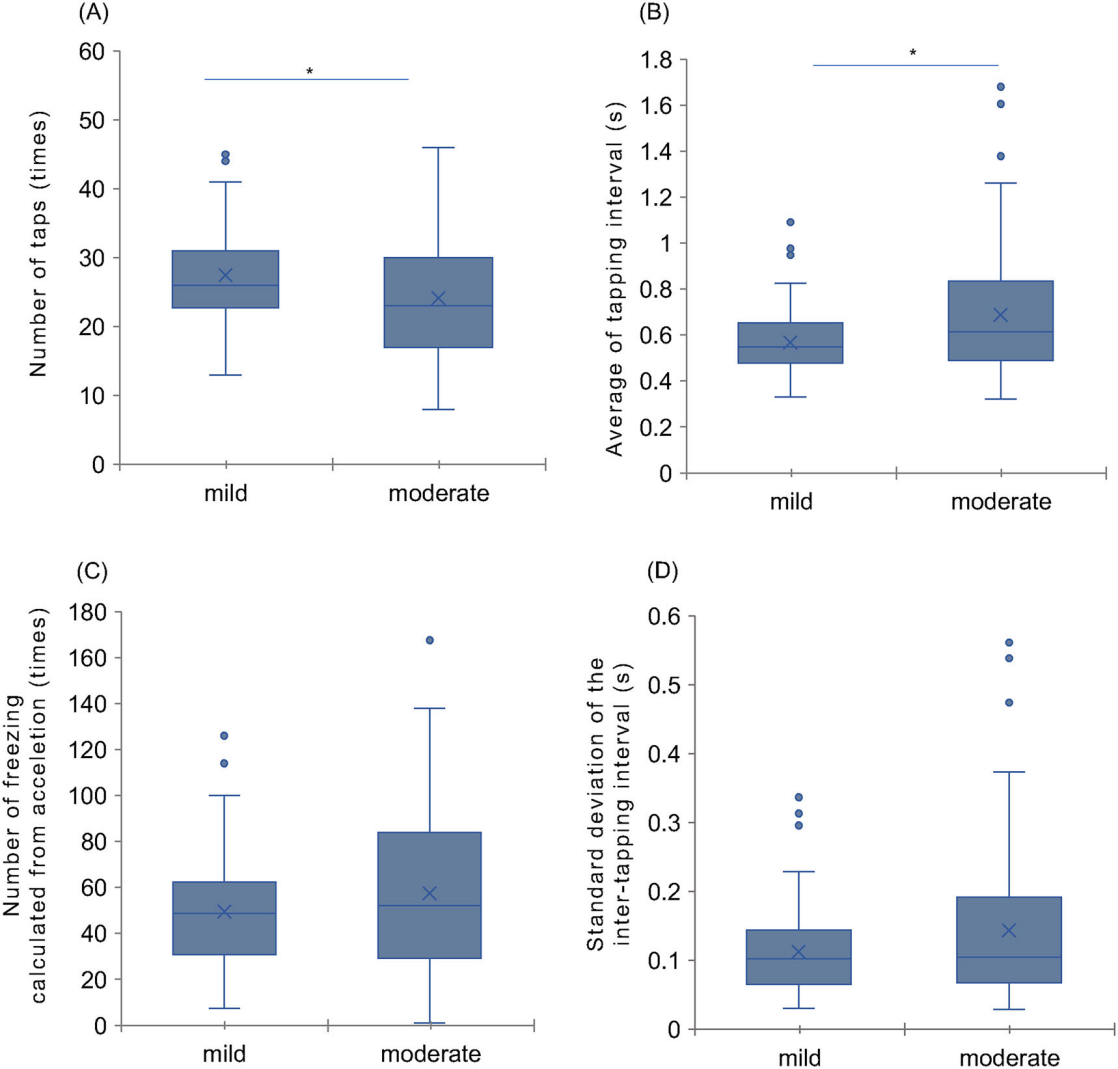
Comparison of finger‐tapping parameters in the mild and moderate AD groups. Compared with the mild AD group, the moderate AD group shows fewer taps and a longer interval between the thumb and index finger and tapping interval. *p < 0.05. Description of finger‐tapping parameters. (A) Number of taps: number of taps during the measurement period. (B) Average of tapping interval: the average intertapping interval between one tap and the subsequent tap.

The comparison of finger parameters between the mild and moderate AD groups revealed significant differences in the number of taps (*p* = 0.005; *r* = 0.22) and average of tapping interval (*p* = 0.007; *r* = 0.21). Compared with the mild AD group, the moderate AD group showed fewer taps and a longer interval between the thumb and index finger and tapping interval. No significant differences were found in the other parameters (standard deviation of the inter‐tapping interval and number of freezing calculated from acceleration). Figure 3 shows the finger parameters that were significantly different. The details of these results are presented in Table S1

### 3.2 Relationship Between Finger‐Tapping Parameters and the MMSE Score

**FIGURE 4:**
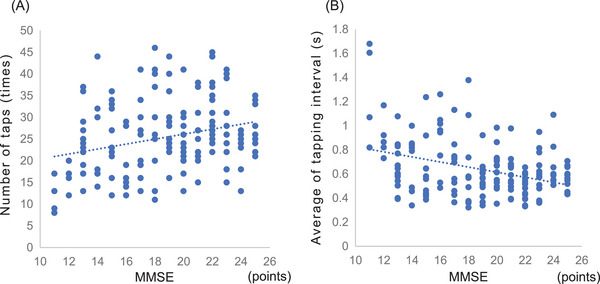
Relationship between finger‐tapping parameters and MMSE scores. Cognitive function (MMSE scores) is correlated with finger function. The number of taps is weakly positively correlated. The average of tapping interval shows weak negative correlations. MMSE, Mini‐Mental Examination.

The correlation between the MMSE score and finger‐tapping parameters was weakly positive for the number of taps (*r* = 0.28, *p* < 0.002) and weakly negative for the average of tapping interval (*r* = −0.26, *p* < 0.001) (Figure 4).

## 4 Discussion

This study compared finger function between patients with mild and moderate AD and showed significant differences in specific motor parameters. The results indicated that the moderate AD group showed fewer taps and a longer tapping interval than the mild AD group, suggesting a distinction in motor performance that aligns with cognitive impairment severity. Additionally, an investigation into the relationship between finger function and cognitive performance revealed weak positive correlations in the number of taps and weak negative correlations in the average of tapping interval.

Although cognitive dysfunction, especially memory impairment, is the most commonly reported symptom of AD, patients with AD also have finger motor function problems. For example, patients have been shown to have reduced finger dexterity and inaccuracy in drawing tasks and pegboard tests compared with controls (Yu and Chang 2016; Suzumura et al. 2022). In addition, delayed response times (Gorus et al. 2008), reduced tapping speed, decreased number of taps, and longer tapping intervals have also been reported (Suzumura et al. 2016). A relationship between motor function and decreased ADL/IADL abilities has also been noted (Lee 2020). Furthermore, in a study comparing finger function between healthy elderly patients and those with MCI, a moderate effect size of *r* ≥ 0.4 was observed for parameters such as the number of taps and tap interval (Suzumura et al. 2022). This study hypothesized that finger dexterity would progressively deteriorate with AD severity. Our findings support this hypothesis, as significant differences were observed in the number of taps and the average of tapping interval between patients with mild and moderate AD. However, it is crucial to interpret these results cautiously owing to the small effect sizes (*r* = 0.21–0.22). These effect sizes suggest that while the differences are statistically significant, the magnitude of these differences is modest. Therefore, finger tapping alone may not be a robust standalone tool for distinguishing between mild and moderate AD. Our findings suggest that finger tapping might be used as a quick screening tool that could potentially improve diagnostics, but the joint sensitivity remains to be studied. Future research should explore whether integrating tapping performance with cognitive assessments could improve overall diagnostic accuracy.

Significant differences were observed in the number of taps and the average of tapping interval between the mild and moderate AD groups. However, no significant differences were found in the standard deviation of the intertapping interval or the number of freezing calculated from acceleration. The average of tapping interval, influenced by motor speed, showed differences between the groups, whereas rhythm, as indicated by intertapping variability, appeared to be preserved across the stages of AD. While the rhythm is not disrupted in mild and moderate AD, the results suggest that tapping speed may decrease with AD severity. The number of freezing calculated from acceleration indicates the number of small freezes other than tapping and the number of times the finger movement temporarily stops or loses momentum during tapping. The reason why there was no significant difference in the number of freezing calculated from acceleration is believed to be that it is less sensitive to the severity of AD compared with indices such as tapping speed that reflect a decline in overall motor function. Previous studies, such as those by Koppelmans et al. have examined unimanual and bimanual finger‐tapping abilities in individuals with MCI and AD, and they showed associations between these motor functions and AD biomarkers (Koppelmans et al. 2023). This finding underscores the importance of finger‐tapping assessments in populations with AD. Additionally, Koppelmans et al. investigated the utility of combining multiple motor measures to improve patient classification accuracy, suggesting that integrating various motor assessments may enhance diagnostic precision (Koppelmans et al. 2024). These studies suggest the potential benefits of a multifaceted motor evaluation to distinguish AD severity levels more effectively.

The present study investigated the relationship between finger function and cognitive function, finding a weak positive correlation between the number of taps and a weak negative correlation between the average of tapping interval. Previous research has shown that patients with AD generally exhibit longer contact duration, slower tapping speed, and fewer taps than healthy older adults, supporting our findings (Suzumura et al. 2016). While this study identified specific motor characteristics associated with AD severity, the neurophysiological mechanisms underlying these changes were not directly investigated. Future research could focus on the roles of specific brain regions, such as the corpus callosum (Kennerley et al. 2002), motor areas, and cerebellum (Dhamala et al. 2003; Théoret et al. 2001; Jacobs et al. 2018; Tabatabaei‐Jafari et al. 2017), which contribute to motor and cognitive integration.

Previous studies have similarly identified relationships between physical performance and cognitive function, such as the link between executive function and the TUG test result (McGough et al. 2011) and the reduction in gait speed during dual tasks compared to single tasks (Montero‐Odasso et al. 2009). Additionally, previous studies have reported correlations between finger dexterity, executive function, and attentional function in patients with dementia (Rudd et al. 2023). This implies that cognitive impairments, such as planning and movement coordination (executive function) or attention, may influence motor performance. Our cross‐sectional findings complement these studies by demonstrating that specific motor parameters may reflect cognitive function. Future longitudinal research could further clarify these relationships over time.

This study has some limitations. First, on the basis of previous studies (Perneczky et al. 2006), we excluded patients with MMSE scores of ≥26 to focus on patients with mild‐to‐moderate cognitive decline. However, it is possible that individuals with scores of ≥26 could still meet the diagnostic criteria for AD. Excluding individuals with scores >26 may have limited our sample to those with more apparent cognitive impairment, potentially restricting our assessment of motor function changes in the early stages of AD. Future studies should consider including individuals with a broader range of MMSE scores to evaluate motor function changes across all stages of AD, including the early stages. Second, the severity of cognitive function was judged only by the MMSE score, highlighting the importance of assessing not only the MMSE score alone but also attention and executive functions to comprehensively understand cognitive function. Third, we could not follow the progress of the patients because this was a cross‐sectional study. Longitudinal follow‐up is needed to confirm the changes in finger function and cognitive function in patients with dementia. Fourth, although this study's results suggest that the finger‐tapping test may help assess AD severity, given the lack of sensitivity and specificity assessments, the finger‐tapping test alone may not be sufficient. Future studies should evaluate these measures alongside other cognitive and motor function tests. Fifth, age, sex, and education level were not adjusted for in this study's analysis. Although these factors did not significantly differ between the mild and moderate AD groups, they may exert differential effects within each group. The lack of adjustments for these factors may weaken the strength of our findings, as age, sex, and education level could each affect motor and cognitive functions. Future studies would benefit from a more sophisticated statistical approach, incorporating these factors to refine the understanding of their potential impact within each AD severity group. Lastly, all findings in this study were based on left‐hand performance in right‐handed individuals aged ≥65 years, which may limit the generalizability of our results to left‐handed individuals and younger populations. Hand dominance can influence motor performance and coordination, potentially affecting tapping ability and interhand asynchrony. Additionally, motor and cognitive function changes in AD may vary in younger patients, possibly affecting tapping performance and the motor‐cognitive relationship. Future studies should explore left‐hand function in left‐handed individuals and include younger participants to assess these patterns across a broader range of ages and hand dominance.

## 5 Conclusions

Our study findings suggest that finger function differs between mild and moderate stages of AD, with significant variations observed in tapping speed and tapping interval. These findings may contribute to the development of comprehensive assessment tools that integrate finger parameters with existing clinical evaluations for AD.

## Author Contributions

**Shota Suzumura**: conceptualization, data curation, formal analysis, investigation, writing–original draft, funding acquisition. **Aiko Osawa**: writing–review and editing. **Junpei Sugioka**: data curation, formal analysis, investigation. **Masaki Kamiya**: writing–review and editing. **Yuko Sano**: conceptualization, software. **Akihiko Kandori**: conceptualization, software. **Tomohiko Mizuguchi**: conceptualization, software, resources. **Yoshiharu Uchida**: conceptualization, software, resources. **Hitoshi Kagaya**: writing–review and editing, supervision. **Izumi Kondo**: conceptualization, writing–review and editing, supervision, project administration.

## Ethics Statement

All the participants or their family members were provided with detailed verbal and written descriptions of the experiments. This study was approved by the ethics committee of our hospital (approval number: 623‐12) and conducted in accordance with the Declaration of Helsinki guidelines.

## Conflicts of Interest

Coauthors Yuko Sano and Akihiko Kandori are employees of Hitachi Ltd. Tomohiko Mizuguchi and Yoshiharu Uchida are employees of Maxell Ltd., but they were not involved in the data collection and analysis of this study. The National Center for Geriatrics and Gerontology conducts joint studies with Hitachi Ltd. and Maxell Ltd. The device used in this study was loaned to the National Center for Geriatrics and Gerontology by Maxell Ltd. The other authors report no relevant disclosures.

### Peer Review

The peer review history for this article is available at https://publons.com/publon/10.1002/brb3.70403.

## Supporting information



Supplementary Figure 1. Points to note during the measurement

Supplemental Table 1. Results of finger tapping (anti‐phase left hand)

## Data Availability

The data supporting the findings of this study are available on request from the corresponding author. The data are not publicly available due to privacy or ethical restrictions.
