# Supplementary material for: Differences in Finger Dexterity in Patients With Mild and Moderate Alzheimer's Disease—A Study of Cognitive Function by Disease Severity
Source: Brain Behav. 2025 Mar 9;15(3):e70403. doi: 10.1002/brb3.70403 (PMC11891264; doi:10.1002/brb3.70403)
Supplement: Supplementary file 1 — Supplementary Figure 1. Points to note during the measurement [file BRB3-15-e70403-s002.docx]

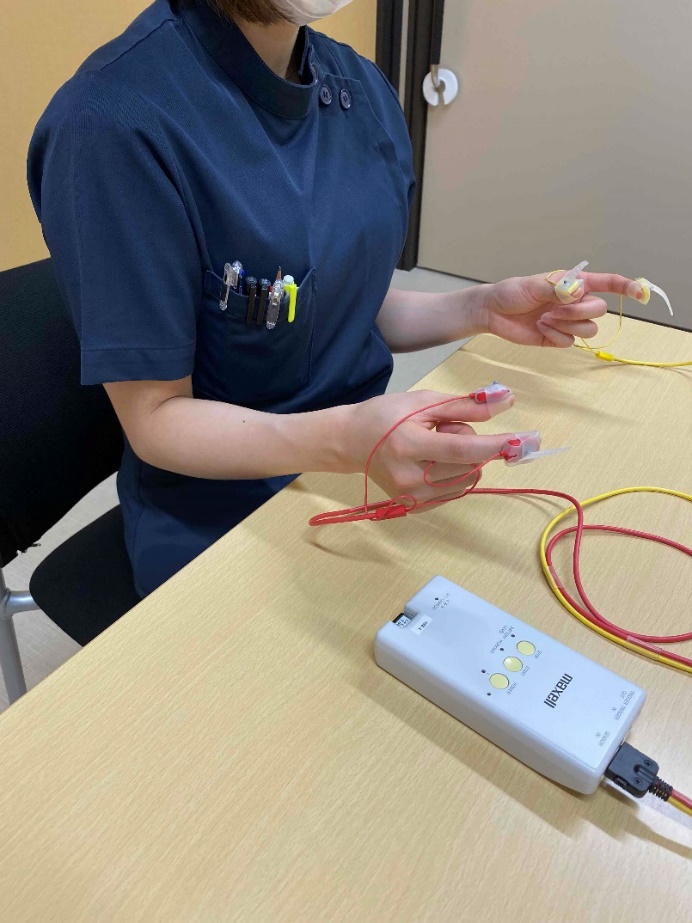


**Supplementary Figure 1. Points to note during the measurement**

1. Keep the elbow joint off the desk.
2. Keep the forearms in the intermediate position between pronation-supination and the upper arms close to the body.
3. Keep the wrist joints in slight dorsiflexion.
4. Hold the third to fifth fingers lightly.
