# Supplementary material for: Differences in Finger Dexterity in Patients With Mild and Moderate Alzheimer's Disease—A Study of Cognitive Function by Disease Severity
Source: Brain Behav. 2025 Mar 9;15(3):e70403. doi: 10.1002/brb3.70403 (PMC11891264; doi:10.1002/brb3.70403)
Supplement: Supplementary file 2 — Supplemental Table 1. Results of finger tapping (anti‐phase left hand) [file BRB3-15-e70403-s001.docx]

**Supplemental Table 1. Results of finger tapping (anti-phase left hand)**

| Parameter | Median  (1st Quartile to 3rd Quartile) | | p-value | Effect size (r) |
| --- | --- | --- | --- | --- |
|  | Mild AD | Moderate AD |  |  |
| Number of taps (times) | 26 (22–31) | 23 (17–30) | **0.005** | 0.22 |
| Ave of tapping interval (s) | 0.56 (0.47–0.65) | 0.61 (0.49–0.84) | **0.007** | 0.21 |
| Number of freezing calculated from acceleration (times) | 49 (30–63) | 52 (29–84) | 0.250 | 0.09 |
| SD of inter-tapping interval (s) | 0.10 (0.06–0.14) | 1.63 (1.19–2.04) | 0.207 | 0.10 |

Abbreviations: AD: Alzheimer’s disease; ave: average; SD, standard deviation
